# Effects of adding low-dose ketamine to etomidate on serum cortisol levels in critically ill cardiac patients: a randomized clinical trial

**DOI:** 10.1186/s12871-022-01654-0

**Published:** 2022-04-21

**Authors:** Mostafa Mohammed Elhamamsy, Ahmed Mohammed Aldemerdash, Fathi Badie Zahran, Gehan Fawzy Mahmoud Ezz, Sara Abou AlSaud, Maged Labib Boules, Mahdy Ahmed Abdelhady, Mohamed Ahmed Hamed

**Affiliations:** 1 King Saud University, Riyadh, Saudi Arabia; 2 Department of Anesthesiology, Faculty of Medicine, Zagazig University, Zagazig, Egypt; 3 Department of Anesthesiology, Faculty of Medicine, Fayoum University, Fayoum, 63511 Egypt

**Keywords:** Critically ill cardiac patients, Acute anemia, Upper endoscopy and Colonoscopy, Etomidate, Ketamine, Serum Cortisol

## Abstract

**Background:**

Etomidate was associated with an inhibition of adrenal steroid synthesis. This study aimed to evaluate the effects of adding low-dose ketamine to etomidate to minimize the decrease in serum cortisol level in critically ill cardiac patients.

**Methods:**

Sixty adult cardiac patients, ≥ 18 years, who underwent upper endoscopy and Colonoscopy to manage acute anemia in the cardiac intensive care units were enrolled. Patients were randomly divided into two groups: (group (E): *n =* 30) received etomidate 0.2 mg/kg IV followed by etomidate 0.05 mg/kg IV, and (group (KE): *n =* 30) received ketamine 0.5 mg/kg IV, then etomidate 0.1 mg/kg IV, followed by etomidate 0.05 mg/kg IV. The primary outcome was Serum cortisol level at 6 h after the procedure.

**Results:**

The mean postoperative cortisol level was significantly lower in group E (295.60 ± 49.218 nmol/L) versus group KE (461.00 ± 67.946 nmol/L), with 95% CI = 351.94 to 404.66; *p =* 0.000. In addition, the estimated serum cortisol reduction level was also significant between groups; In group E, the estimated cortisol level decreased nearly 53% from 632.40 ± 35.066 nmol/L to 295.60 ± 49.218 nmol/L 6 hours postoperative. While in group KE, the estimated cortisol level decreased only 27% from 639.13 ± 43.035 nmol/L to 461.00 ± 67.946 nmol/L.

**Conclusions:**

Single-dose ketamine (0.5 mg/kg) was helpful to decrease the total dose of etomidate and hence decreased the percentage of serum cortisol level in such critically ill patients with preservation of patient satisfaction.

**Trial Registration:**

This study is registered on ClinicalTrials.gov (NCT04857450; principal investigator: Mostafa Mohammed Elsaid Elhamamsy; registration date: 23/04/ 2021).

## Introduction

Gastrointestinal (GI) bleeding is frequently seen in critically ill cardiac patients in the intensive care unit (ICU) due to many causes, including the toxicity of anticoagulation therapy, stress ulceration, chronic peptic ulcers, or neoplasms [1]. Sometimes, the management involves a gastroscopy or a colonoscopy, procedures that should be performed under sedation or general anesthesia. Hemodynamic stability is crucial in this specific cardiac population, and various anesthesia agents could be used.

Etomidate possesses unique, desirable properties such as rapid onset and short duration of action. In addition, it produces less apnea than barbiturates or propofol, no histamine release, infrequent allergic reactions, relative cardiovascular and respiratory stability, and neuroprotective effects, making it an attractive induction agent to facilitate this procedure [2, 3]. However, etomidate side effects also exist, including pain on injection, myoclonic movements during induction of general anesthesia, and postoperative nausea and vomiting [4]. Furthermore, one of the most dangerous etomidate toxicity among anesthetic drugs is the dose-dependent inhibition of adrenal steroid synthesis that far outlasts its hypnotic action, and that may reduce survival of critically ill patients [5]. It has been shown that in patients undergoing elective surgery, cortisol response to surgery was absent 48 h after administering a single bolus of etomidate [6].

Ketamine is a potent, multimodal dissociative anesthetic. It is one of the well-known N-methyl-D-aspartate receptor (NMDA) antagonists [7]. for several years, NMDA receptors were involved in the physiologic regulation of hormones released from the hypothalamic-pituitary-adrenal axis [7]. Several clinical trials have demonstrated that ketamine is associated with increased postoperative serum cortisol levels [7–9]. Low-dose ketamine (0.5 mg/kg) pretreatment was successfully used to reduce the Incidence and severity of etomidate-induced myoclonus [10]. However, no available data about its role in controlling etomidate-induced adrenal suppression. So, we aimed to evaluate the efficacy of low-dose ketamine pretreatment to etomidate on serum cortisol levels in anesthesia for critically ill cardiac patients.

We hypothesized that adding low dose ketamine to etomidate could be helpful to minimize the decrease in serum cortisol level in critically ill cardiac patients in comparison to a regimen including only etomidate.

## Methods

This prospective, randomized, blind clinical trial was performed following the tenets of the Declaration of Helsinki. Institution review board (IRB), King Saud University, Saudi Arabia approval was obtained (Ref. No. 21/0047/IRB), and written informed consent was acquired from all patients. The study protocol was registered at ClinicalTrials.gov. (NCT04857450; principal investigator: Mostafa Mohammed Elsaid Elhamamsy; date of registration: April 23/04/ 2021, no protocol amendment or study changes after trial start). This study adheres to the applicable CONSORT guidelines.

The study included sixty cardiac patients admitted to the cardiac intensive care units at King Saud University scheduled for Esophagogastroduodenoscopy and Colonoscopy to diagnose and manage acute anemia. Including patients aged 18 – 65 years, the American Society of Anesthesiologists (ASA) III-IV, with ejection fraction (EF) > 30%. The Exclusion criteria were as follow: poor left ventricular function (EF < 30%), recent myocardial infarction (last 7 days), known allergy to midazolam, fentanyl, etomidate, or ketamine, severe respiratory (pneumonia or congestive heart failure), hepatic, and renal failure, or history of neurological disorders (Seizure at the onset with postictal residual neurological impairments) or convulsions.

The study was performed in the ICU with all emergency equipment (Ventilators, Monitor with defibrillator with external pacing facility, and Syringe pumps). Patients were randomly divided into two groups (E; etomidate and KE; ketamine/etomidate groups) using computer-generated random numbers placed into separate opaque envelopes opened by the study investigator just before performing the procedure. The gastroenterologist who performed the colonoscopies, all participants, and data collectors were blinded with group allocation till the end of the study. All patients received a standard colonic preparation protocol and were fasted 8 h before the procedures.

### Anesthesia management

Standard monitoring was performed after establishing IV access (noninvasive blood pressure, ECG, SpO2, and [Bispectral Index (BIS) {BIS Complete Monitoring System P/N 185-0151 Covidien IIc, 15 Hampshire Street, Mansfield, MA 02048 USA} maintained below 60]. First, a nasal cannula for oxygen (6 l/min) was connected to all patients. Then midazolam 0.03 mg/kg IV and fentanyl one μg/kg IV were injected in all patients as a premedication.

For (group (E):***n =*** 30) Patient Received Etomidate 0.2 mg/kg IV over 30 s, followed by 0.05 mg/kg IV and repeated when needed manual.

For (group (KE):***n =*** 30) Patient Received ketamine 0.5 mg/kg IV over 30 s, then etomidate 0.1 mg/kg IV over 30 s, followed by 0.05 mg/kg IV and repeated when needed manual.

The anesthesiologist determined the additional dose of etomidate to achieve a BIS above 60. All patients were breathing spontaneously.

Any adverse effect was recorded, including; 1- Hypotension: decreased baseline systolic blood pressure > 30%, or decreased baseline diastolic blood pressure > 30%, treated with phenylephrine 100-200 μg IV boluses. 2- Bradycardia: decrease of HR < 50/min,treated with atropine 0.5 mg IV. 3- Apnea: spontaneous breathing > 30 s, or SpO2 < 85%,treated by assisted manual ventilation using an AMBU bag and face mask.

The total etomidate dose, the duration of the procedure, and the patients’ recovery time were recorded.

After full recovery and when the patients were alert enough to express their attitude regarding the intra-procedural events, they were asked to score their level of satisfaction during the procedure to recall any painful or other undesirable intra-procedural events.

Patient’s satisfaction level was assessed with a Likert five-item scoring system [11]: (1 = Not satisfied at all, 2 = slightly satisfied, 3 = somewhat satisfied, 4 = very satisfied, and 5 = extremely satisfied). Finally, serum cortisol levels both before and 6 h after the procedure were measured to calculate the percentage of suppression in both groups.

The primary outcome was; 6 h postoperative serum cortisol level.

Secondary outcomes included: Estimated change in serum cortisol level (6-hours postoperative value compared to preoperative one), Incidence of perioperative complications; hypotension, bradycardia, apnea, and nausea/vomiting. Patient satisfaction and total dose of etomidate.

### Statistical Analysis

For sample size calculation, there were no previous studies at the time of designing the study protocol, so we performed an external pilot study that included seven patients in each group, with its results not included in the full-scale study. This pilot showed 6 h postoperative serum cortisol level (mean ± SD, 356.28 ± 39.343 in the E group versus 389.71 ± 45.76 in the KE group). The minimal sample size of patients was 27 in each group needed to get power level 0.80 and alpha level 0.05. the calculated sample size was increased by 10% to reach 30 in each group to overcome the data dropout.

The collected data were organized, tabulated, and statistically analyzed using SPSS software statistical computer version 22 (SPSS Inc., USA).

We used a two-sample t-test to compare the two groups’ mean values (age, weight, and Serum cortisol level) and data presented as Mean, standard deviation (SD). And The Chi-square test was used to analyze independent qualitative data. Fischer’s test was used when chi-square test conditions were not met. Data were presented as numbers and percentages (Sex, ASA, and the Side effects), and (95% CI) were estimated. A two-sided *P-*value of < 0.05 was considered statistically significant.

## Results

**Fig. 1:**
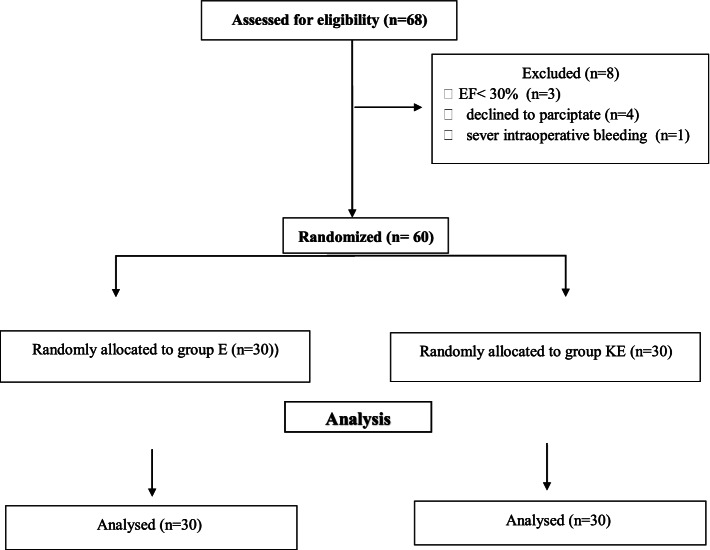
Consort flow diagram of the study population

For this study, 68 patients were assessed for eligibility based on the inclusion and exclusion criteria. Eight patients have excluded; three cases with EF below 30%, four patients declined to participate, and one patient developed severe intraoperative bleeding from esophageal varices and was intubated to avoid aspiration. The remaining 60 patients were randomly assigned into the study groups **(**Fig. 1**).**

The demographic characteristics and operative data concerning age, sex, body weight, ASA physical status, the length of the procedure, and the time needed for recovery were similar between the two groups **(**Table 1**)**.

**Table 1:** Demographic characteristics and operative data

	Group E	Group KE	***P***-value ^**a,b**^
**Sample size,n**	30	30	
**Mean age (SD) in (years)**	49 (11.59)	50.07 (11.20)	0.756
**Mean Weight (SD) in kg**	78.70 (8.774)	81.13 (8.905)	0.415
**Sex, n (%)**			
**Male**	17 (56.66)	16 (53.33)	0.795
**Female**	13 (43.33)	14 (46.66)	
**ASA, n (%)**			
**III**	27 (90)	26 (86.66)	0.688
**IV**	3 (10)	4 (13.33)	

*Abbreviations: SD* Standard deviation, *N* Number, *Group E* Etomidate group, *Group KE* Ketamine Etomidate group, *ASA* American Society of Anesthesiologists

^**a**^ *P-*value compares group E versus the group KE

^**b**^ Mann-Whitney and Chi-square test used was used to analyze data

**Fig. 2:**
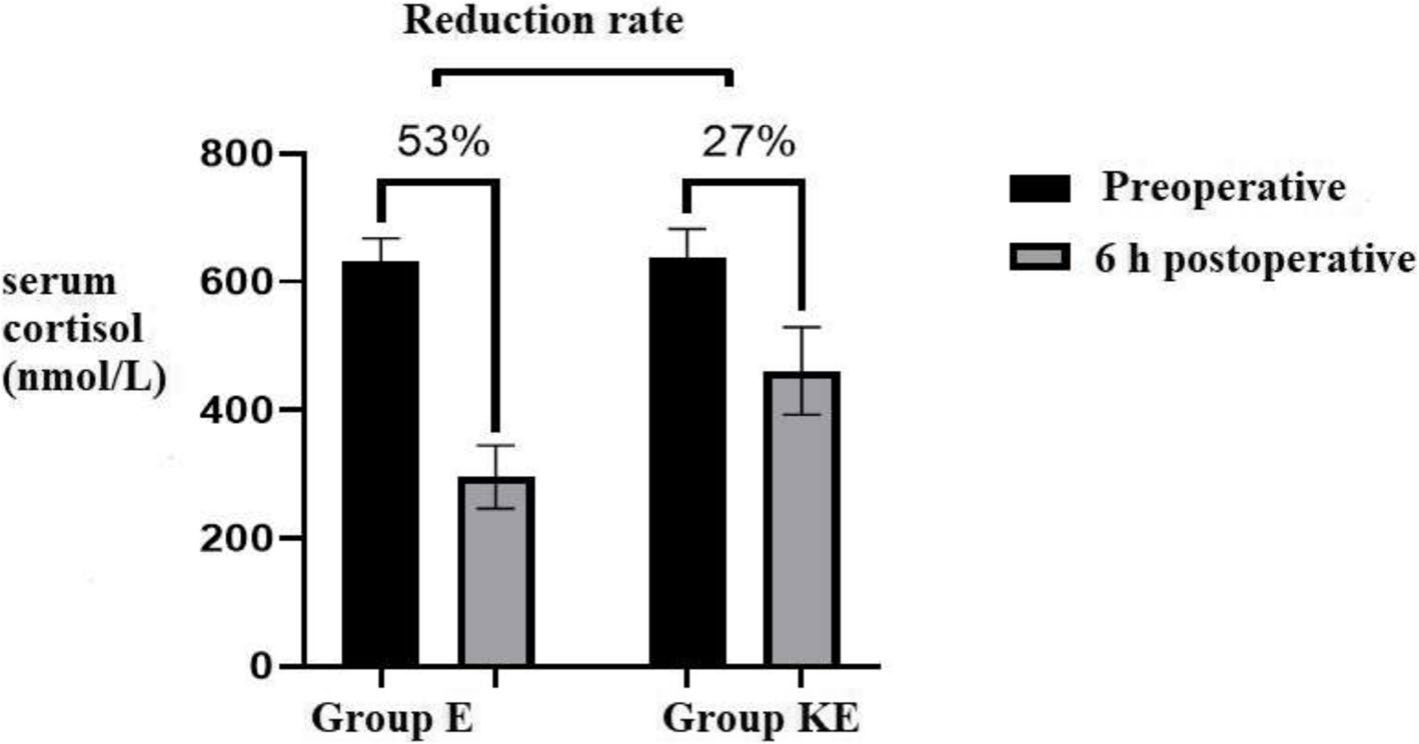
Serum cortisol level pre-and 6 h postoperative

The mean 6 hours postoperative serum cortisol level significantly reduced in group E than group KE; (295.60 ± 49.218) nmol/L versus (461.00 ± 67.946) nmol/L respectively With 95%CI = 351.94 to 404.66; *p =* 0.000 **(**Table 2**)**. In addition, the estimated serum cortisol reduction level was also significant between groups; In group E, the estimated cortisol level decreased nearly 53% from 632.40 ± 35.066 nmol/L before the procedure to 295.60 ± 49.218 nmol/L 6 hours after the procedure. While, in group KE, the estimated cortisol level decreased only 27% from 639.13 ± 43.035 nmol/L before the procedure to 461.00 ± 67.946 nmol/L 6 hours after the procedure (*p* < 0.05) **(**Fig. 2**).**

**Table 2:** Serum cortisol level pre-and 6 h postoperative

	Group E	Group KE	95%CI	***P***-value ^**a,b**^
**Sample size, n**	30	30		
**Mean Preoperative cortisol (SD) in (nmol/L)**	632.40 (35.066)	639.13 (43.035)	625.67 to 645.86	0.728
**Mean 6 h Postoperative cortisol (SD) in (nmol/L)**	295.60 (49.218)	461.00 (67.946)	351.94 to 404.66	0.000

*Abbreviations: SD* Standard deviation, *N* Number, *Group E* Etomidate group, *Group KE* Ketamine Etomidate group, *CI* Confidence Interval

^a^ *P*-value compares group E versus the group KE

^b^ Two samples t-test used to compare means

The total dose of etomidate was significantly different between the two groups being 34.10 ± 5.095 mg in group E versus 23.93 ± 4.346 mg in Group KE (*P* < 0.001)**.**

There was no significant difference between the groups regarding patient satisfaction (Table 3). In addition, there was no significant difference between the groups regarding the Incidence of adverse effects during the procedure (Table 4).

**Table 3:** Patient satisfaction

	Group E	Group KE	95% CI	***P***-value ^a,b^
**Sample size,n**	30	30		
**Patient Satisfaction, n (%)**				
**Slightly Satisfied**	3(10%)	5(16.66%)	4.09 to 4.44	
**Very Satisfied**	13(43.33%)	15(50%)		0.520
**Extremely Satisfied**	14(46.66%)	10(33.33%)		

*Abbreviations: N* Number, *Group E* Etomidate group, *Group KE* Ketamine Etomidate group, *CI* Confidence Interval

^a^ *P*-value compares group E versus the group KE

b Chi-square test used was used to analyze data

**Table 4:** The adverse events during the procedure

	Group E	Group KE	95%CI	***P***-value ^a,b^
**Sample size,n**	30	30		
**Hypoxia, n (%)**	5(16.66%)	2(6.66%)	0.03 to 0.20	0.228
**Bradycardia, n (%)**	5(16.66%)	2(6.66%)	0.03 to 0.20	0.228
**Hypotension, n (%)**	5(16.66%)	2(6.66%)	0.03 to 0.20	0.228
**Nausea and Vomiting, n (%)**	2(6.66%)	0(0.0%)	−0.01 to 0.08	0.150

*Abbreviations: N* number, *Group E* Etomidate group, *Group KE* Ketamine Etomidate group, *CI* Confidence Interval

^a^ *P*-value compares group E versus the group KE

^b^ Chi-square test used was used to analyze data

## Discussion

Our study shows that serum cortisol levels were reduced in both groups at 6 h postoperatively compared to preoperative levels. However, there was a marked reduction in the etomidate group than the ketamine/etomidate group, which was statistically significant. The difference in cortisol we observed could result from giving ketamine or giving less etomidate, and this trial cannot distinguish between the two. These findings meet that A. K. Pandey et al. [8] reported that serum cortisol levels decrease significantly after etomidate administration while increasing significantly after ketamine administration.

Etomidate is well known to decrease serum cortisol levels. Several reports in the literature have described adrenocortical suppression after single-dose administration of etomidate with reversibility in normal healthy subjects [12–14]. On the other hand, N. Hergovich et al. [7] reported a significant increase in serum cortisol after ketamine administration in healthy volunteers. Furthermore, ketamine infusion has been described as a dose-dependent increase in serum cortisol level [9].

Etomidate-induced adrenocortical suppression could hinder survival in critically ill patients [10]. On the other hand, increased serum cortisol after ketamine administration worsens surgical-induced stress response [8]. So, we successfully used minimal-dose ketamine as a pretreatment to etomidate to counteract its effect on serum cortisol. Low-dose ketamine pretreatment to etomidate was used safely and successfully to reduce etomidate-induced myoclonus [10] But, to our knowledge, no available studies about the role of ketamine in controlling etomidate-induced adrenocortical suppression.

The mechanism of anesthetic effects on the adrenocortical function and steroidogenesis remains unclear. However, etomidate-induced adrenocortical suppression has been explained by directly inhibiting the enzymes involved in cortisol biosynthesis, especially the final enzyme in the cascade, 11 β-hydroxylase [15]. In their animal study, Besnier E et al. [15] described an effect of ketamine on the hypothalamic-pituitary-adrenal axis similar to that of etomidate. However, no clinical evidence supports these findings.

Also, we found no significant difference between both groups regarding some adverse events (hypotension, bradycardia, apnea, and nausea/vomiting) or patient satisfaction, with favorable outcomes. These findings support the hypothesis elicited by the authors that low-dose ketamine pretreatment to etomidate can successfully counteract the impact of etomidate on serum cortisol without additional side effects or aggravation of stress response. This finding is met with Wu GN et al. [10] as they found that ketamine reduced the Incidence and severity of myoclonus without any additional adverse effects.

Based on the current study’s findings described by Wu GN et al. [10] low-dose ketamine pretreatment to etomidate could be used safely and has favorable outcomes than etomidate alone. However, survival at day 7 is more remarkable with ketamine [16]. This finding elicits a question about adding ketamine to etomidate to make a new preparation (Ketamine-etomidate) that can achieve the benefits of both drugs without any additional adverse effects.

Bruder et al. [17] and Albert et al. [18] reported that etomidate causes adrenal insufficiency; it was not shown to increase mortality in critically ill patients.

In our study, The total dose of etomidate was significantly different between the two groups. We were collecting the total dose of Etomidate However, the dose of etomidate as varied as the induction dose in the preplanned protocol because we show the effect of the low dose of ketamine and decrease of the total amount of etomidate in cortisol level and patient satisfaction.

### Limitations

First, the limit of our study was the short time follow-up (only 6 h), so we could not test the reversibility of serum cortisol to baseline values after anesthesia and hospital and ICU stay and mortality rate assessment. Also, our study population was specific with unique characteristics; all are critically ill cardiac patients with acute anemia, which could limit our data’s generalizability. Furthermore, the study is not powered for rare adverse events and several other adverse events related to etomidate and ketamine. And visual analog scores were not assessed as we depend on patient satisfaction only. In addition, lack of available data for comparison as this is the first clinical trial testing the effect of low-dose ketamine pretreatment to etomidate on postoperative cortisol levels. Despite these limitations, our study highlights helpful in decreasing the total dose of etomidate and diminishing the serum cortisol level after ketamine administration and low dose Etomidate.

## Conclusions

Single-dose ketamine (0.5 mg/kg) was helpful to decrease the total dose of etomidate and hence decreased the percentage of serum cortisol level in such critically ill patients with preservation of patient satisfaction.

## Data Availability

The datasets used and analyzed during the current study are available from the corresponding author on reasonable request.

## Abbreviations

ASA: The American Society of Anesthesiologists
BIS: Bispectral Index
CI: Confidence Interval
E: Etomidate
EF: Ejection fraction
GI: Gastrointestinal
KE: Ketamine/etomidate
ICU: Intensive care unit
IRB: Institution review board
NMDA: N-methyl-D-aspartate receptor
SD: Standard deviation
