# Correction: Effects of adding low‑dose ketamine to etomidate on serum cortisol levels in critically ill cardiac patients: a randomized clinical trial

**DOI:** 10.1186/s12871-023-01971-y

**Published:** 2023-01-13

**Authors:** Mostafa Mohammed Elhamamsy, Ahmed Mohammed Aldemerdash, Fathi Badie Zahran, Gehan Fawzy Mahmoud Ezz, Sara Abou AlSaud, Maged Labib Boules, Mahdy Ahmed Abdelhady, Mohamed Ahmed Hamed

**Affiliations:** 1 King Saud University, Riyadh, Saudi Arabia; 2 Department of Anesthesiology, Faculty of Medicine, Zagazig University, Zagazig, Egypt; 3 Department of Anesthesiology, Faculty of Medicine, Fayoum University, Fayoum, 63511 Egypt

**Correction: BMC Anesthesiol 22, 114 (2022)**

**https://doi.org/10.1186/s12871-022-01654-0**

Following publication of the original article [1], the authors reported an error to the affiliation of authors Maged Labib Boules and Mahdy Ahmed Abdelhady. Both authors should affiliated to "Fayoum University" and not "Zagazig University". The correct affiliation is "Department of Anesthesiology, Faculty of Medicine, Fayoum University, Fayoum 63511, Egypt".

The original article [1] has been updated.
